# Perceived Benefit and Satisfaction With a Tablet Computer and an Emergency Smartwatch by Older Adults and Their Relatives: Prospective Real-World Pilot Study

**DOI:** 10.2196/53811

**Published:** 2024-08-02

**Authors:** Patrick Wiegel, Marina Liselotte Fotteler, Brigitte Kohn, Sarah Mayer, Filippo Maria Verri, Dhayana Dallmeier, Michael Denkinger

**Affiliations:** 1 Research Unit on Ageing, Agaplesion Bethesda Clinic Ulm, Zollernring 26, Ulm, 89073, Germany, 49 731187 ext 191; 2 Institute for Geriatric Research, Ulm University Medical Center, Ulm, Germany; 3 Geriatric Center Ulm, Ulm, Germany; 4 DigiHealth Institute, Neu-Ulm University of Applied Sciences, Neu-Ulm, Germany; 5 Department of Epidemiology, Boston University School of Public Health, Boston, MA, United States

**Keywords:** assistive technology, older adults, caregiver, benefits, usability, gerontechnology

## Abstract

**Background:**

Assistive technologies (ATs) have the potential to promote the quality of life and independent living of older adults and, further, to relieve the burden of formal and informal caregivers and relatives. Technological developments over the last decades have led to a boost of available ATs. However, evidence on the benefits and satisfaction with ATs in real-world applications remains scarce.

**Objective:**

This prospective, real-world, pilot study tested the perceived benefit and satisfaction with different ATs in the real-world environment.

**Methods:**

Community-dwelling adults aged ≥65 and their relatives tested a tablet computer with a simplified interface or a smartwatch with programmable emergency contacts for 8 weeks in their everyday life. Perceived benefits and satisfaction with ATs were assessed by all older adults and their relatives using different assessment tools before and after the intervention. Outcome measures included the Technology Usage Inventory, Quebec User Evaluation of Satisfaction with Assistive Technology 2.0, and Canadian Occupational Performance Measure.

**Results:**

A total of 17 older adults (tablet computer: n=8, 47% and smartwatch: n=9, 53%) and 16 relatives (tablet computer: n=7, 44% and smartwatch: n=9, 56%) were included in the study. The number of participants that were frail (according to the Clinical Frailty Scale) and received care was higher in the smartwatch group than in the tablet computer group. Older adults of the smartwatch group reported higher technology acceptance (Technology Usage Inventory) and satisfaction (Quebec User Evaluation of Satisfaction with Assistive Technology 2.0) scores than those of the tablet computer group, although the differences were not significant (all *P>*.05). In the tablet computer group, relatives had significantly higher ratings on the item *intention to use* than older adults (*t*_12.3_=3.3, *P*=.006). Identified everyday issues with the Canadian Occupational Performance Measure included contact/communication and entertainment/information for the tablet computer, safety and getting help in emergency situations for the smartwatch, and the usability of the AT for both devices. While the performance (*t*_8_=3.5, *P*=.008) and satisfaction (*t*_8_=3.2, *P*=.01) in these domains significantly improved in the smartwatch group, changes in the tablet computer group were inconsistent (all *P>*.05).

**Conclusions:**

This study highlights the remaining obstacles for the widespread and effective application of ATs in the everyday life of older adults and their relatives. While the results do not provide evidence for a positive effect regarding communication deficits, perceived benefits could be shown for the area of safety. Future research and technical developments need to consider not only the preferences, problems, and goals of older adults but also their relatives and caregivers to improve the acceptability and effectiveness of ATs.

## Introduction

In their global disability action plan from 2015, the World Health Organization (WHO) defined assistive technologies (ATs) as “any item, piece of equipment or product, whether it is acquired commercially, modified or customized, that is used to increase, maintain or improve the functional capabilities of individuals with disability” [1]. For older adults, ATs can positively impact not only functionality but also autonomy, safety, and communication. In particular, ATs might support older adults who wish to remain in their own homes instead of depending on institutional care. In addition, the application of ATs has become a valid option in relieving the formal and informal caregivers of older adults [2].

In recent years, the field of ATs has evolved to include a wide range of devices for different audiences, varying in complexity and price. Examples include mobile health applications [3], wearable devices [4,5], robotic systems [6], virtual reality applications [7], or sensory aids [8]. ATs are used for various purposes such as personal disease management [3], managing fall risks [9], ensuring correct medication [10,11], and preventing social isolation [12,13]. Clearly, there is an abundance of available ATs assisting with age-related challenges. Yet, the effectiveness of ATs for older adults remains inconclusive [3,9,12,14]. Nevertheless, studies investigating the benefits and usability of ATs for older adults in the real-world environment are still lacking [15-17]. A focus on multimorbid and frail populations is of special interest, as one would expect that this cohort might especially benefit from the use of ATs. In addition, the use of ATs by older frail and nonfrail adults often depends on and includes relatives and caregivers who significantly contribute to the (successful) application of ATs. Additionally, this group itself might benefit from the application of ATs through a reduction of care burden [18]. Thus, studies testing the effectiveness of ATs should also consider the effects on caregivers and relatives.

Older adults are less likely than younger adults to use technology, and this depends on a number of factors including sociodemographic factors, attitudinal variables, and cognitive abilities [19]. However, despite the assumption that older adults are afraid and reluctant to use ATs, recent evidence indicates that they are relatively open to the idea of using technologies in their everyday life, independent of their age and subjective health status [20,21]. The factors that influence the intention of older adults to use digital technologies are complex and include environmental, psychological, and social determinants [22]. Among the most important ranked criteria for the selection and use of ATs by older adults are the promotion of independence, affordability, ease of use, and ethical company policies [23]. Thus, the use and effectiveness of ATs ultimately depend on the alignment of the technological developments and user needs.

Recently, emergency buttons or watches and wearable devices with GPS tracking or fall detection for older adults have gained special interest. However, few studies exist investigating the benefit of these devices for older adults. Most existing research focuses on wearables worn for disease monitoring or activity tracking [24]. A recent study with an emergency button (worn as a wristband or necklace) that was connected to the landline did not show any improvement for health-related quality of life and other outcome measures [5]. Focusing on the issues of loneliness and lack of social interaction, software systems for (tablet) computers designed specifically for older adults are entering the market. The systems feature large icons and simple menus. Functionalities include video calls, access to the news, photo albums, or games. However, the benefit of such ATs remains unclear. While 1 study reported a significant improvement of loneliness, social support, and well-being among community-dwelling older adults living alone using a special computer system [13], a large systematic review and meta-analysis of similar interventions found no benefits on psychological outcomes in people with cognitive impairment and dementia [7].

This prospective, real-world, pilot study aims to assess the individual benefits and satisfaction gained from an emergency smartwatch and a senior tablet computer by community-dwelling older adults and their relatives in the real-world environment. The products were selected based on the results of a product competition for companies.

## Methods

### Study Population

Since the use of ATs often requires or supports the contact and interaction with relatives, this study considered 2 participant groups: older adults and their relatives or a close friend (hereinafter referred to as relatives). The inclusion criteria for older adults were (1) aged ≥65 years; (2) residence in the area of Ulm (distance ≤50 km); (3) an independent or assistive living situation; (4) the ability to speak and read German; and (5) the ability to consent to the study participation (adults with dementia were excluded). In addition, older adults had to have intact vision and hearing (normal or corrected). The relatives had to (1) be a family member or someone close to the adult; (2) speak and read German; (3) give consent to study participation; and (4) have a smartphone, tablet computer, or PC with internet connection. Participants were recruited in Southern Germany using convenience (via analogous and digital recruitment methods such as flyers, social media, and clinic staff) and snowball sampling (asking participants for other potential participants). As this was an exploratory study, no sample size calculation was conducted. The aim was to recruit between 6 to 10 participants per selected device. This number was defined based on feasibility. For 4 different ATs with 5 available devices each, 2 rounds of the study with 40 participants can be performed within 4 months.

### Ethical Considerations

Ethical approval was obtained from Ulm University Ethical Committee (Nr. 230/21, 05.07.2021). Before entering the study, individuals received detailed information about the study and provided written informed consent to participate. Data analyses were performed on pseudonymized data. No financial remuneration was provided for participation.

### Selection of the ATs

Initially, 4 different AT were chosen for this interventional study. The ATs were selected from the 2020 product competition “Daheim Dank Digital” (at home thanks to digitalization) [25] aimed at startups and established companies in German-speaking countries (Germany, Austria, and Switzerland). Companies were asked to apply with ATs that could compensate age-related deficits and impairments described in 5 predefined use cases. The use cases were designed together with experts from different areas of expertise and focused on (1) nocturnal restlessness and fall risk; (2) loneliness, hearing and vision impairment, and forgetfulness; (3) inactivity and listlessness in daily routine; (4) urinary incontinence and reduced fluid and food intake; and (5) limited mobility, weakness, and loneliness.

A total of 9 companies presented their products, and a scientific jury evaluated the devices based on availability; readiness for use; and impact in at least 1 of the categories of communication, security, or autonomy. These categories were specified as target areas in the overall project. A total of 4 products were selected for this user study based on their availability. The identified devices focused on safety (an intelligent bed exit alarm from the company NevisQ and a smartwatch with an emergency button from the company CareIOT GmbH) and communication (the Media4Care tablet computer from Media4Care GmbH and the Eldertech app, which supports communication and coordination of care within a family, from Eldertech GmbH). At the time of the study, all products were commercially available and had a CE certificate.

### Study Design

We performed a prospective, real-world, pilot study of 4 different ATs with community-dwelling adults aged ≥65 years and their relatives. Together with the participants, the investigators decided which product was the most suitable for the older adult. Based on their individual needs, a decision was made for 1 of the targeted areas: communication or safety. Unfortunately, no candidates were identified for the evaluation of the bed exit alarm and the app; therefore, the selection was limited to the tablet computer and the emergency smartwatch. Both devices were used in their versions that were current as of August 2021. Details on the main functionalities of these 2 devices are listed in Table 1. Further technical information on the 2 products can be found in Multimedia Appendix 1.

In an introductory assessment session, all participants and their relatives were informed of the study procedure, goals, and possible risks and consented to participate. Baseline data were captured in a subsequent meeting at the participant’s home or in the study center (SM and BK). Participants were given the selected AT and a short introduction to the device. Participants then tested the AT in their daily life over a period of 8 weeks. In case problems with the use of the AT arose, participants were asked to first seek help in the manual, on the web, or via the company hotline before contacting the study center. This was done to reproduce a real-life setting as accurately as possible.

During the study period, the study team called the participants after 1, 2, 4, and 6 weeks to inquire about the use of the product; help solve possible issues; and remind participants to document errors, problems, and the frequency of use. After the test period of 8 weeks, the participants and their relatives were assessed a second time. This meeting took place at the participant’s home or in the study center. The relatives performed an independent assessment of the AT based on their user experience gathered during the testing period (assisting or interacting with older adults). Thus, the relatives’ ratings represent their own perception of the AT rather than the perception of the usefulness of the AT for the older adult.

**Table 1.** Main functionalities of the 2 assistive technologies tested in the study.

Device	Main functionalities and features
Smartwatch	Emergency button, including the notification of contacts via phone and mail GPS tracking Analysis of movement profile and automatic notification of contact persons in case of deviations from the norm
‍Tablet computer	Video calls Messenger service Entertainment (games, news, podcasts, radio, and music) Photo album Touchscreen

The questionnaires were paper based and filled out by 1 of the investigators (SM or BK) during the interview sessions before and after the intervention. Older adults and their relative were interviewed in separate sessions to reduce the interview duration and to avoid interaction, except when older adults required support from the relative. Interviews were performed face-to-face with all older adults. Interviews with relatives were also performed by phone.

### Data Collection

At baseline, sociodemographic data were collected including sex, age, living situation (alone, together with partner, or with someone else), education (<10 y or ≥10 y), level of care (administratively assigned level of care measuring a person’s care need and determining their claim for additional support), self-perceived health status (excellent, very good, good, fair, or poor), and self-perceived age. Additionally, information on interest in technology was collected (high, medium, or low).

To assess older adults’ ability to perform instrumental activities of daily living (IADL), the Lawton scale was used [26]. The corrected IADL score was calculated taking into account the possibility that the activities have never been performed [27]. To capture additional information about the participants’ social situation, the Lubben Social Network Scale-6 was used [28]. With the Clinical Frailty Scale (CFS), frailty and fitness of the older adults (scale from 1=“very fit” to 9=“terminally ill”) were assessed [29]. In addition, both older adults and relatives had to rate their life satisfaction (scale from 0 to 10, with higher values indicating higher life satisfaction).

#### Frequency of Use

Older adults were asked to document their use frequency of the technology in each of the 8 intervention weeks. The frequency of use was reported on a 5-point scale, that is, it captured whether the technology was used on 7, 5‐6, 3‐4, 1‐2, or 0 days per week.

#### Technology Usage Inventory

The Technology Usage Inventory (TUI) was administered to the older adults and their relatives to assess the influence of psychological factors on the use and acceptance of technology [30]. The items *curiosity* and *technology anxiety* were asked before the intervention, whereas the items *interest*, *usability*, *usefulness*, *skepticism*, and *accessibility* were asked after the intervention. Answers were given on a 7-point Likert scale (1=“strongly disagree” and 7=“strongly agree”). In addition, the *intention to use* the AT was asked on a visual analog scale (0=“agree” and 10=“disagree”) after the intervention. For orientation purposes, the item *intention to use* has been reversed in the *Results* section of this paper.

#### Quebec User Evaluation of Satisfaction With Assistive Technology 2.0

Satisfaction with the technology was evaluated after the intervention using the German version of the Quebec User Evaluation of Satisfaction with Assistive Technology (QUEST) 2.0 [31]. Of the original 12 items of the QUEST 2.0, only the 8 items relating to an AT device (domain device) were considered. Each question was scored on a 5-point scale (1=“not satisfied at all” and 5=“very satisfied”). The questionnaire was completed by both the older adults and their relative. Additionally, they were asked to choose the 3 items with the highest relevance for them. Overall satisfaction was calculated as the mean score across all answered items. The QUEST 2.0 has been shown to be a valid and reliable assessment for a population of assistive device users [32].

#### Canadian Occupational Performance Measure

The Canadian Occupational Performance Measure (COPM) is a semiquantitative, client-centered instrument that was used to investigate the perceived satisfaction with the performance of the AT in the specified problem areas of the older adults and their relatives [33]. For this purpose, everyday issues of personal importance related to the use of the AT were determined together with an occupational therapist during the interview before the intervention. For each participant, up to 5 everyday issues were identified, and for each of these issues, the older adults and their relatives had to rate their perceived level of performance and satisfaction on a scale from 1 to 10, with higher scores indicating higher performance or satisfaction. Ratings for the defined issues were then summed and divided by the number of issues to obtain a performance score and a satisfaction score. After testing the AT, the older adults and their relatives repeated the rating on the previously defined everyday issues of personal importance.

### Data Analysis and Statistics

For all outcome measures, descriptive statistics were calculated and included the mean (SD). The focus of the analysis was on the outcomes that assessed the usability aspects of the ATs, that is, the TUI and QUEST 2.0. In addition, exploratory statistical inference testing was performed to assess the effects of the ATs on the specified everyday issues of the older adults and their relatives (COPM). The assumption of a normal distribution was tested by the Kolmogorov-Smirnov test of normality. Subsequently, 2-tailed *t* tests were used to compare intervention groups using the Welch *t* tests (life satisfaction, TUI, and QUEST 2.0) and preintervention and postintervention data using paired *t* tests (COPM). Uncorrected *P* values are presented in the *Results* section. However, to control the false discovery rate, we used the correction of the *P* values via the Benjamini-Hochberg procedure [34]. If this correction changed the outcome of the statistical test, this is reported in the *Results* section. In addition, effect sizes were calculated using Cohen *d*. The statistical significance was set at *P*<.05 for all tests. Statistical analyses were performed using RStudio (RStudio Team).

## Results

### Participants

The study was performed from August 2021 to April 2022. A total of 44 older adults were screened for inclusion in the study. From this sample, 18 older adults and their relatives met the inclusion criteria and were enrolled in this study. There was 1 dropout who stopped study participation due to dissatisfaction with the technology. A total of 17 older adults (tablet computer: n=8, 47% and smartwatch: n=9, 53%) and 16 relatives (tablet computer: n=7, 44% and smartwatch: n=9, 56%) completed the study and were included in the final data analysis. In the tablet computer group, there were 2 cases where the same relative belonged to 2 older adults, and there was 1 case where 2 relatives belonged to the same older adult.

The sociodemographic characteristics of the older adults and their relatives are shown in Tables2  3, respectively. Older adults in both groups were aged >80 years on average but reported a younger self-perceived age. Even though participants in the smartwatch group were older on average, they felt younger than those in the tablet computer group. Data on self-perceived health status, level of care, corrected IADL, and frailty indicated that older adults in the smartwatch group were more dependent and slightly frailer than those in the tablet computer group. A total of 4 participants, all in the smartwatch group, perceived their own health to be poor or fair. However, older adults of the smartwatch group were socially more engaged than those of the tablet computer group. While most older adults (tablet computer: 7/8, 88% and smartwatch: 6/9, 67%) and all relatives indicated at least a medium level of technology interest, one-third (3/9, 33%) of participants in the smartwatch group reported a low level of technology interest. Relatives in the smartwatch group had a higher mean age and included more male persons than the relatives in the tablet computer group.

**Table 2.** Sociodemographic characteristics of older adults. Data are presented as frequency and percentage or as mean (SD).

Characteristics		Tablet computer (n=8)	Smartwatch (n=9)
**Sex, n (%)**			
	Female	5 (62)	5 (56)
	Male	3 (38)	4 (44)
Age (years), mean (SD)		80.1 (8.2)	82.7 (7.9)
Self-perceived age (years), mean (SD)		76.9 (12.4)	72.6 (15.4)
**Living situation, n (%)**			
	Alone	3 (38)	6 (67)
	With partner	5 (62)	3 (33)
**Education (years), n (%)**			
	<10	4 (50)	4 (44)
	≥10	4 (50)	5 (56)
**Self-perceived health status, n (%)**			
	Excellent or very good	2 (25)	1 (11)
	Good	6 (75)	4 (44)
	Fair or poor	0 (0)	4 (44)
**Level of care** ^**a**^ **, n (%)**			
	Yes	2 (25)	5 (56)
	No	6 (75)	4 (44)
Corrected IADL^b^, mean (SD)		7.4 (1.4)	5.3 (2.6)
CFS^c^ score, mean (SD)		3.4 (1.2)	3.7 (2.1)
Frailty (CFS score>4), n (%)		0 (0)	4 (44)
LSNS-6^d^ score, mean (SD)		15.4 (3.5)	21.8 (2.0)
Socially isolated (LSNS-6 score<12), n (%)		1 (12)	0 (0)
**Technology interest, n (%)**			
	High	4 (50)	4 (44)
	Medium	3 (38)	2 (22)
	Low	1 (12)	3 (33)

a Level of care: administratively assigned level of care measuring a person’s care need and determining their claim for additional support.

b IADL: instrumental activities of daily living (Lawton scale).

c CFS: Clinical Frailty Scale.

d LSNS-6: Lubben Social Network Scale-6.

**Table 3.** Sociodemographic characteristics of relatives. Data are presented as frequency and percentage or as mean (SD).

Characteristics		Tablet computer (n=7)	Smartwatch (n=9)
**Sex, n (%)**			
	Female	6 (86)	5 (56)
	Male	1 (14)	4 (44)
Age (years), mean (SD)		50.3 (18.2)	64.3 (11.3)
**Education (years), n (%)**			
	<10	1 (14)	1 (11)
	≥10	6 (86)	8 (89)
**Technology interest, n (%)**			
	High	3 (43)	5 (56)
	Medium	4 (57)	4 (44)
	Low	0 (0)	0 (0)

### Frequency of Use

The frequency of use of the tablet computer and smartwatch in everyday life was heterogeneous across the study period. In the tablet computer group, 5 older adults reported varying use frequencies, which ranged between not using the technology at all (0× per week) and regularly using the technology (7× per week). Two participants of the tablet computer group stopped using the technology after the first week of the intervention. In the smartwatch group, 6 older adults used the technology consistently across all 8 intervention weeks (mostly 7× per week). In the smartwatch group, 2 participants stopped using the technology after the first 1 to 3 weeks. A total of 2 older adults (1 in each group) did not document their frequency of use.

### Life Satisfaction

The mean life satisfaction of older adults changed nonsignificantly from 7.9 (SD 3.3) to 7.3 (SD 3.1) in the tablet computer group (*t*_7_=1.1, *P*=.31, *d*=0.29) and from 7.2 (SD 2.1) to 8.1 (SD 1.9) in the smartwatch group (*t*_8_=1.1, *P*=.29, *d*=0.44). Similarly, the mean life satisfaction of relatives changed nonsignificantly from 8.9 (SD 0.9) to 8.0 (SD 2.1) in the tablet computer group (*t*_6_=1.5, *P*=.17, *d*=0.53) and from 7.7 (SD 1.4) to 8.1 (SD 0.7) in the smartwatch group (*t*_6_=0.9, *P*=.41, *d*=0.39). In the smartwatch group, 2 participants only provided the preintervention (n=1) or postintervention (n=1) value and were not considered in the statistical analysis.

### Technology Acceptance

Prior to the intervention, mean ratings for technology acceptance of older adults and their relatives ranged between 5.8 and 6.5 points for *fearfulness* and between 4.4 and 5.1 points for *curiosity* in the tablet computer and smartwatch group, respectively. For both items, the differences between the tablet computer and smartwatch groups were not significant (older adults: *fearfulness P*=.11 and *curiosity P*=.77; relatives: *fearfulness P*=.43 and *curiosity P*=.36). After the intervention, ratings from the older adults and their relatives of the tablet computer and smartwatch groups were similar for the items *interest*, *accessibility*, *usability*, and *skepticism* (older adults: *interest P*=.90, *accessibility P*=.64, *usability P*=.26, and *skepticism P*=.67; relatives: *interest P*=.65*, accessibility P*=.78*, usability P*=.64, and *skepticism P*=.39). This was different for the items *usefulness* and *intention to use*. TUI scores on the item *usefulness* were higher in the smartwatch group (older adults: mean 5.0, SD 1.0; relatives: mean 5.4, SD 1.4) than in the tablet computer group (older adults: mean 3.8, SD 1.5; relatives: mean 4.2, SD 1.6), although this did not reach statistical significance (older adults: *t*_12.3_=1.9, *P*=.07, *d*=0.98; relatives: *t*_12.1_=1.6, *P*=.14, *d*=0.83). TUI scores on the item *intention to use* were significantly higher in the smartwatch group (mean 6.1, SD 3.4) than in the tablet computer group (mean 2.7, SD 2.2; *t*_13.6_=2.4, *P*=.03, *d*=1.14) in older adults (note that after correction for multiple comparisons, the *P* value exceeded .05). Although ratings on *intention to use* from the relatives were also higher in the smartwatch group (mean 7.8, SD 2.7) than in the tablet computer group (mean 5.9, SD 1.5), the differences were not significant (*t*_11.0_=1.7, *P*=.11, *d*=0.86). Interestingly, in the tablet computer group, relatives’ ratings on the item *intention to use* (mean 5.9, SD 1.5) were on average twice as high as those of older adults (mean 2.6, SD 2.2; *t*_12.3_=3.3, *P*=.006, *d*=1.66). In contrast, the relatives’ and older adults’ ratings on the item *intention to use* were similar in the smartwatch group (older adults: mean 6.1, SD 3.4; relatives: mean 7.8, SD 2.7; *t*_14.8_=1.1, *P*=.28, *d*=0.54). Descriptive data on all items of the TUI can be found in Multimedia Appendix 2.

### Satisfaction With the Technology

**Figure 1.**
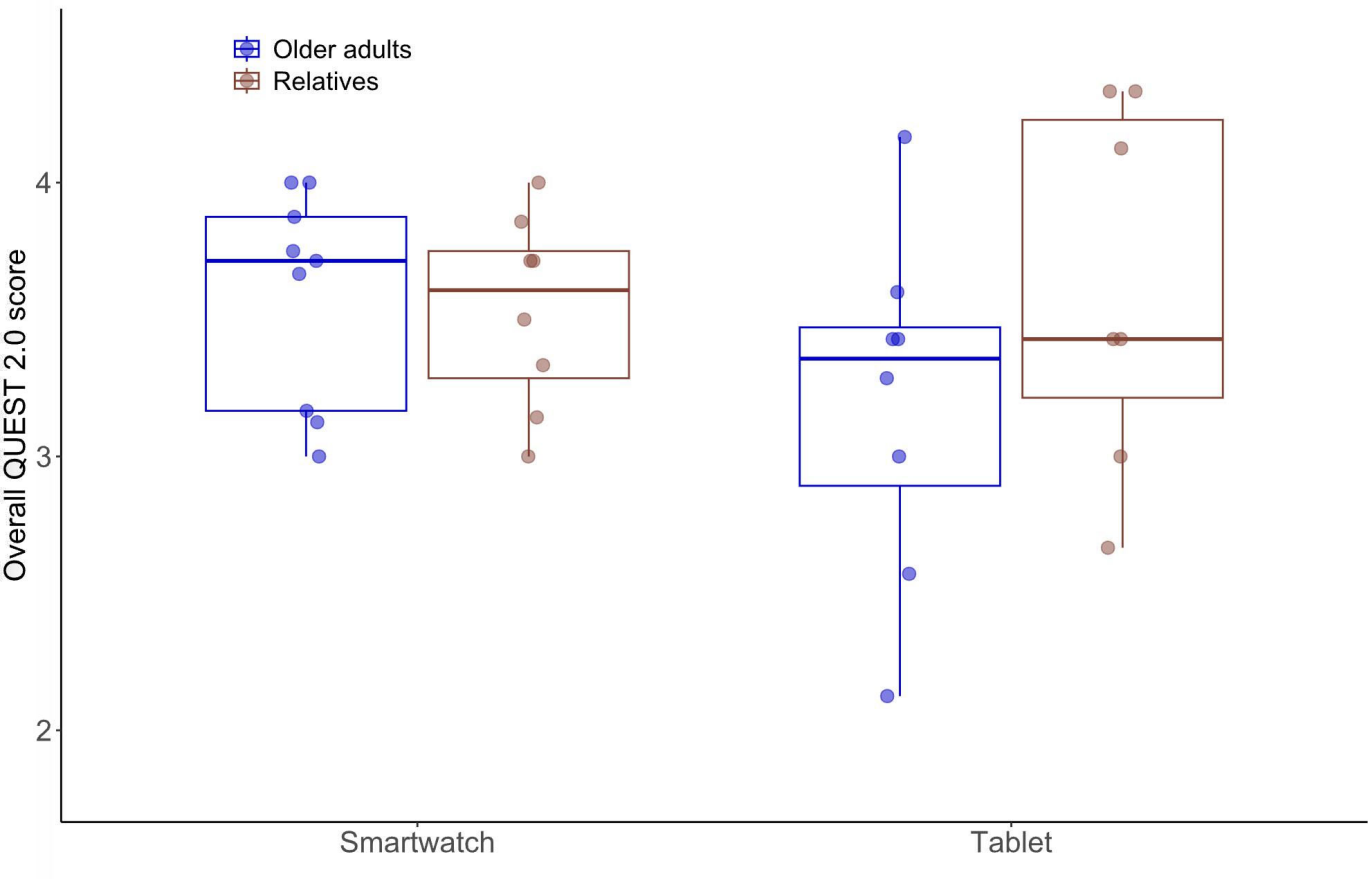
QUEST 2.0 score (scale from 1 to 5) of older adults and their relatives for the tablet computer and smartwatch groups. Higher values represent higher satisfaction. Dots represent data from individual participants. QUEST: Quebec User Evaluation of Satisfaction with Assistive Technology.

The overall satisfaction score of the QUEST 2.0 across all items was similar for the older adults and relatives of both groups (Figure 1). The mean satisfaction score of older adults was 3.2 (SD 0.6) in the tablet computer group and 3.6 (SD 0.4) in the smartwatch group (*t*_11.4_=1.5, *P*=.16, *d*=0.75). Similarly, the mean satisfaction across all items rated by the relatives was 3.6 (SD 0.7) in the tablet computer group and 3.5 (SD 0.4) in the smartwatch group (*t*_8.6_=0.3, *P*=.77, *d*=0.16).

The 3 most relevant rated items for both AT groups were *safety and reliability* (n=4), *ease of use* (n=7), and *effectiveness* (n=7). Thus, these items were analyzed separately (Table 4). This analysis suggested that older adults of the smartwatch groups rated all 3 items better than older adults of the tablet computer group. Likewise, the relatives of the smartwatch group rated the items *safety and reliability* and *effectiveness* better than those of the tablet computer group. This was different for the item *ease of use*. This item was rated better by the relatives of the tablet computer group than those of the smartwatch group. In addition, item ratings of relatives were better than those of older adults in the tablet computer group. However, none of the abovementioned comparisons yielded significant test results (older adults: *safety and reliability P*=.06, *ease of use P*=.57, and *effectiveness P*=.40; relatives: *safety and reliability P*=.21, *ease of use P*=.40, and *effectiveness P*=.21).

**Table 4.** Results (mean and SD) of selected QUEST^a^ 2.0 items (scale from 1 to 5) for the tablet computer and smartwatch groups. Higher values represent higher satisfaction.

QUEST 2.0 items	Tablet computer		Smartwatch	
	Older adults (n=8)	Relatives (n=7)	Older adults (n=9)	Relatives (n=9)
Safety and reliability, mean (SD)	2.8 (1.0)	3.2 (1.5)	3.7 (0.7)	3.5 (1.2)
Ease of use, mean (SD)	3.1 (1.4)	3.9 (1.2)	3.9 (0.8)	3.5 (1.2)
Effectiveness, mean (SD)	3.1 (1.4)	3.2 (1.5)	3.6 (1.5)	3.9 (1.6)

a QUEST: Quebec User Evaluation of Satisfaction with Assistive Technology.

### Self-Perceived Performance and Satisfaction

The most frequently reported everyday issues identified in the COPM were contact/communication with relatives (tablet computer group), entertainment/information on news (tablet computer group), perception of safety (smartwatch group), getting help in emergency situations (smartwatch group), and usability of the AT (both groups). The number of specified issues ranged between 1 and 4.

**Figure 2.**
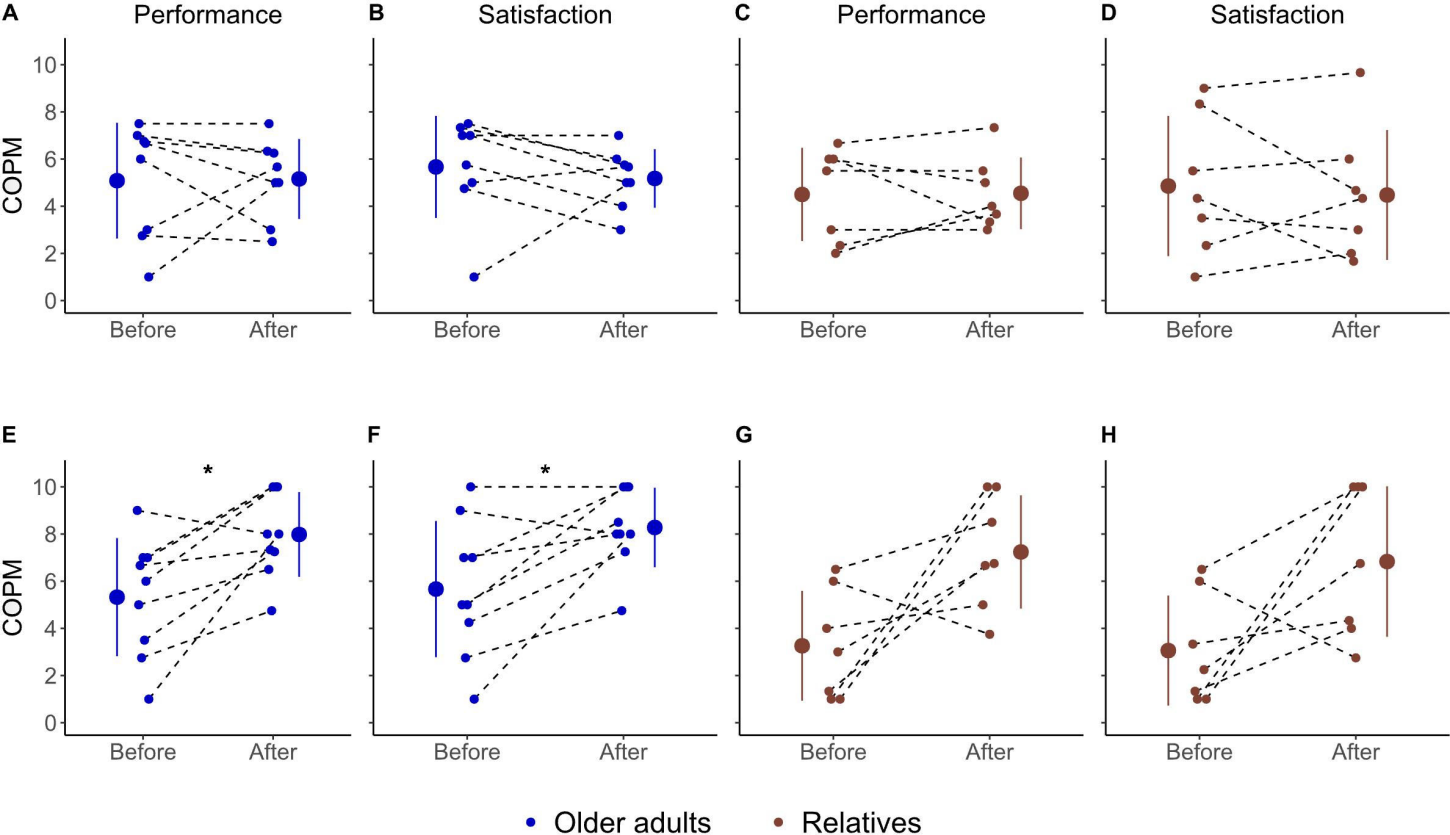
COPM score (scale from 0 to 10) of older adults and their relatives for the (A-D) tablet computer and (E-H) smartwatch groups. Higher values represent higher self-perceived performance and satisfaction. Small dots represent data from individual participants. Bigger dots and error bars represent mean and SD. Asterisks (*) indicate significant differences after correction for multiple comparisons. COPM: Canadian Occupational Performance Measure.

In the tablet computer group, the older adults and their relatives reported diverse scores for self-perceived performance and satisfaction, with both improved and reduced scores. Statistical analysis showed that self-perceived performance (older adults: *t*_7_=0.09, *P*=.93, *d*=0.03; relatives: *t*_6_=0.08, *P*=.94, *d*=0.03) and satisfaction (older adults: *t*_7_=0.6, *P*=.54, *d*=0.23; relatives: *t*_6_=0.5, *P*=.64, *d*=0.18) did not significantly change in the tablet computer group during the intervention (Figure 2A-D). This was different in the smartwatch group. All but 1 older adult and 1 relative reported improved performance and satisfaction in the defined issues. Statistical analysis showed that self-perceived performance (*t*_8_=3.5, *P*=.008, *d*=1.17) and satisfaction (*t*_8_=3.2, *P*=.01, *d*=1.06) significantly increased in the older adults of the smartwatch group (Figure 2E-F). In the relatives of the smartwatch group, self-perceived performance (*t*_6_=2.5, *P*=.04, *d*=0.96) and satisfaction (*t*_6_=2.3, *P*=.06, *d*=0.87) increased, although this result was not statistically significant (after multiple comparison correction; Figure 2G-H).

## Discussion

### Principal Findings

This prospective, real-world, pilot study analyzed the perceived benefit and satisfaction with 2 types of AT for older adults and their relatives addressing different user needs (communication related vs safety related). The devices were (1) a tablet computer with a video call function and entertainment content for older adults and (2) a smartwatch with an emergency button and GPS tracking. Both older adults and their relatives in the smartwatch group consistently reported improved outcomes in the domains of life satisfaction and self-perceived performance and positive ratings for technology acceptance and satisfaction. Several participants emphasized feeling more secure when going out while wearing the device. In contrast, experiences with the tablet computer were more diverse, with several participants reporting poorer technology acceptance, satisfaction, and self-perceived performance.

Wearables with sensor-based risk assessment or fall detection and GPS locating can contribute to reducing older adults’ fear of going outside and thus preserve participation in the community, autonomy, and mobility [35-37]. However, issues with device aesthetics, reliability, and ease of use can negatively impact device acceptance [35,38,39]. Most available studies that focus on the accuracy of device measurements are conducted within a laboratory setting. Contrary to our study, the main sensor location in existing studies is the waist or lower back [36,37]. However, it has been shown that a sensor location at the wrist (eg, watch or wristband) is an important feature for better acceptance of the device [40]. Another study found the highest satisfaction for a pendant worn on a key chain or around the neck [35]. Both forms have the advantage of being familiar or comfortable—aspects that are essential for technology adoption as stated by Fischer et al [41]. Although many emergency smartwatches for older adults have entered the market in recent years, only a few intervention studies with devices similar to the smartwatch tested here exist. Thus, the benefit and results for older adults and their caregivers remain inconclusive [38,42]. Future interventional studies in a real-world setting that take into account the wearers’ wishes and focus on reliability, familiarity, and ease of use are needed.

Tablet computers for older adults are designed to improve participation, provide social support, and reduce loneliness or anxiety. Based on the existing research, the effectiveness of these systems remains inconclusive [5,13,43]. In our study, older adults in the tablet computer group reported on average reduced life satisfaction after the 8-week test period and worse scores for safety and reliability when compared to the smartwatch group. Several participants reported technical failures. Some of the issues mentioned included video calls that did not work, difficulties in receiving pictures from relatives, and defective charging. Considering the high value put on device reliability, these problems are part of the explanation for the negative results of the tablet computer. Additionally, the device is operated via a touchscreen, a technology older adults are mostly unfamiliar with. Consistently, the item ease of use was rated higher by the relatives in the tablet computer group, who belong to a generation already familiar with the use of a touchscreen [43].

Participants received no training prior to using the ATs in their daily lives. This procedure was chosen to create a situation that is as realistic as possible. In most cases, older adults purchasing ATs need to rely on the assistance of their relatives or the company support hotline [35]. In both groups, there were 2 participants who stopped using the ATs after the first few weeks of the intervention period, potentially due to a lack of adequate preparation or lack of support. Indeed, previous research indicates that older adults wish to receive additional training of the technology items they use in the home [44]. Thus, it might be necessary to incorporate training, particularly for more innovative ATs with reduced familiarity and potentially challenging features [38].

This study included 3 older adults characterized as vulnerable or prefrail (CFS score=4) and 4 with some level of frailty (CFS score>4). Physical frailty is a “medical syndrome with multiple causes and contributors that is characterized by diminished strength, endurance, and reduced physiologic function that increases an individual’s vulnerability for developing increased dependency and/or death.” (p. 4) [45]. Frail older adults face specific challenges when it comes to using and deriving benefits from ATs [46]. However, this subgroup is frequently underrepresented in intervention studies, thereby impeding a comprehensive understanding of their requirements [47]. In this study, all vulnerable or frail older adults reported a high likelihood for intention to use the ATs, indicating that this population is overall open to using ATs in their daily lives. Additionally, vulnerable or frail older adults had an average technology satisfaction score of 3.5 (SD 0.5), that is, their satisfaction was between “more or less satisfied” and “quite satisfied.”

As clearly deduced from the introductory WHO definition [1], the term AT is a very broad umbrella term, and it covers an extremely heterogeneous group of products. Each product targets users with specific needs and specific goals. Measuring such diversity is challenging within scientific research. The COPM is unique in the sense that it allows the analysis and quantification of individual user needs and specific AT aims. Thus, it can be adapted to different settings, targets, domains, and participants—irrespective of their age, sex, or other attributes. The tool has been used in other studies investigating the effectiveness of ATs [48,49]. The TUI and QUEST 2.0, however, include predefined items or questions that are not developed specifically for older adults. A recent study suggests that independence, affordability, ease of use, and ethics are the most important AT evaluation criteria for older adults [23]. It is possible that these domains are not adequately represented by the TUI and QUEST 2.0. Assessments focusing on older adults or specifically adapted to the needs and wishes of this group might be better suited in future studies.

### Limitations

The 2 ATs studied target different domains (communication vs safety). Thus, the comparability and generalizability of results are limited. However, the used of the COPM allowed an item-specific evaluation according to its properties to help resolve the identified issues. Although the overall interest in the study was high, only 17 older adults and 16 relatives were included in this study. Thus, the statistical power of the study was relatively low, and further studies with larger samples are required to draw robust conclusions. Having the participation of a relative as a prerequisite for enrollment was one of the biggest obstacles for recruitment as they often declined to participate due to time restrictions. However, as many ATs require or support the interaction with relatives, we consider it important to investigate both perspectives, that of the older adults and that of the relatives. A large systematic review on ATs for older adults with dementia analyzed 571 studies and found that most investigations of clinical effectiveness were conducted with small sample sizes of <20 participants [50]. Conducting real-life intervention studies on the effectiveness of ATs requires a significant amount of effort and time. Combined with difficult recruitment among the population aged ≥65 years, this explains the low sample sizes. Unfortunately, we did not find any older adults–caregiver dyads who were willing to test the smart bed rail or the care planning app and able to participate in the study assessments. Both products are possibly of interest for frail older adults with a higher risk for fall or need for care. These individuals might be too sick to participate in a study such as this, live in some form of assisted living facilities or nursing homes, and be more difficult to reach. Older adults with no interest in technology did not participate in the study, causing a certain sample bias. The study was conducted with the device versions (including the software) that were current as of August 2021. The reproduction of the study using newer device versions could potentially change the results.

### Conclusion

This prospective, real-world, pilot study confirmed the potential of ATs to support older adults and their relatives, especially for safety-related issues, but also highlighted the remaining obstacles for widespread use. Frail older adults and their relatives, who would potentially benefit the most from ATs, are especially difficult to reach. Thus, future research and technical developments of ATs should take into account the preferences, problems, and goals of older adults. In addition, this study highlights that individualized measures such as the COPM are necessary to identify the needs and assess the user benefits of the ATs in real-world applications.

## Supplementary material

10.2196/53811 Multimedia Appendix 1 Technical Information on tablet and smartwatch.

10.2196/53811 Multimedia Appendix 2 Results (mean and SD) of the Technology Usage Inventory (TUI, scale from 1 to 7 for all items except for intention to use (scale from 1 to 10)) for the tablet and smartwatch group. Higher values represent better evaluations.

## Abbreviations

AT: assistive technology
CFS: Clinical Frailty Scale
COPM: Canadian Occupational Performance Measure
IADL: instrumental activities of daily living
QUEST: Quebec User Evaluation of Satisfaction with Assistive Technology
TUI: Technology Usage Inventory
WHO: World Health Organization
