# Supplementary material for: Perceived Benefit and Satisfaction With a Tablet Computer and an Emergency Smartwatch by Older Adults and Their Relatives: Prospective Real-World Pilot Study
Source: JMIR Hum Factors. 2024 Aug 2;11:e53811. doi: 10.2196/53811 (PMC11310738; doi:10.2196/53811)
Supplement: Multimedia Appendix 1 [file humanfactors-v11-e53811-s001.pdf]

Multimedia Appendix 1: Technical Information on tablet and smartwatch.

	**Tablet**	**Smartwatch**
**Company**	Media4Care GmbH, Berlin, Germany	CareIOT GmbH, Schwalmstadt, Germany
**Product**	Media4care (model: Samsung Galaxy Tab A7)	Cares.Watch Profi
**Size**	10.4 " (2000 x 1200 pixel)	46x38x15mm (without wristband)
**Software**	Android, OS version 10, App version 5.16.5	Sim card + Web-Application
**CE**	yes	yes
**Website (only available in German)**	https://www.media4care.de/	https://www.cares.watch/
