# Supplementary material for: Perceived Benefit and Satisfaction With a Tablet Computer and an Emergency Smartwatch by Older Adults and Their Relatives: Prospective Real-World Pilot Study
Source: JMIR Hum Factors. 2024 Aug 2;11:e53811. doi: 10.2196/53811 (PMC11310738; doi:10.2196/53811)
Supplement: Multimedia Appendix 2 [file humanfactors-v11-e53811-s002.pdf]

Multimedia Appendix 2: Results (mean ± SD) of the Technology Usage Inventory (TUI, scale from 1 to 7 for all items except for intention to use (scale from 1 to 10)) for the tablet and smartwatch group. Higher values represent better evaluations.

	Tablet		Smartwatch	
	Older adults	Relatives	Older adults	Relatives
Prior to intervention				
Fearfulness	5.8 ± 1.2	6.2 ± 0.9	6.5 ± 0.4	5.9 ± 0.8
Curiosity	4.7 ± 0.9	5.1 ± 1.6	4.8 ± 0.8	4.4 ± 1.0
After intervention				
Interest	4.9 ± 1.6	5.3 ± 1.3	5.0 ± 1.4	5.6 ± 1.3
Accessibility	5.4 ± 1.1	5.3 ± 1.3	5.6 ± 1.3	5.5 ± 1.3
Usability	4.9 ± 0.9	4.4 ± 0.9	5.4 ± 0.9	4.6 ± 0.7
Usefulness	3.8 ± 1.5	4.2 ± 1.6	5.0 ± 1.0	5.4 ± 1.4
Scepticism	5.6 ± 1.2	5.7 ± 0.9	5.9 ± 1.4	6.1 ± 0.9
Intention to use	2.7 ± 2.2	5.9 ± 1.5	6.1 ± 3.4	7.8 ± 2.7
